# Supplementary material for: Scope and Limitations of Exploiting the Ability of the Chemosensitizer NV716 to Enhance the Activity of Tetracycline Derivatives against Pseudomonas aeruginosa
Source: Molecules. 2023 May 23;28(11):4262. doi: 10.3390/molecules28114262 (PMC10254829; doi:10.3390/molecules28114262)
Supplement: Supplementary file 1 [file molecules-28-04262-s001.zip › molecules-2395915-supplementary.pdf]

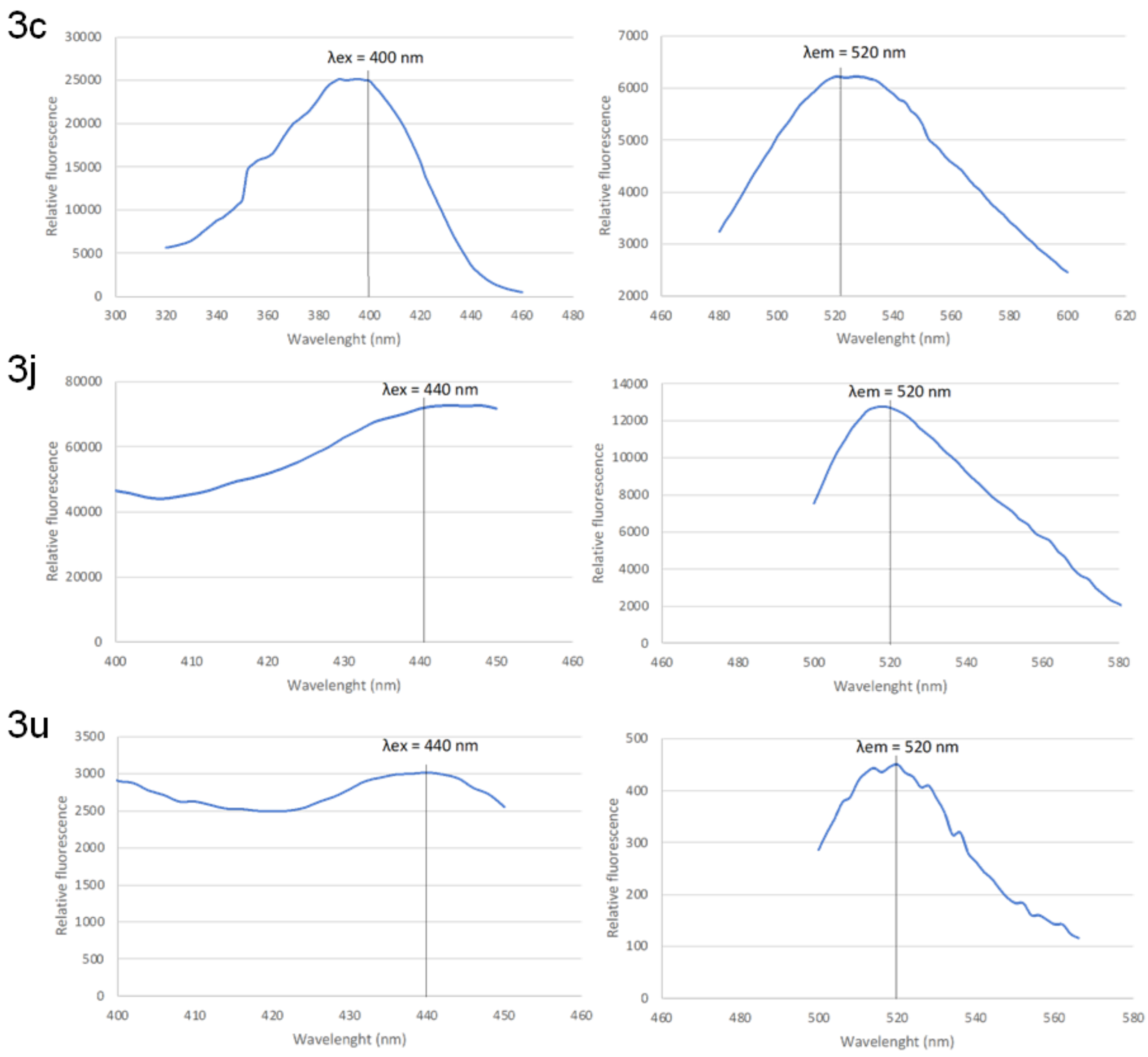

Figure S1. Excitation and emission spectra of compounds **3c**, **3j,** and **3u**.

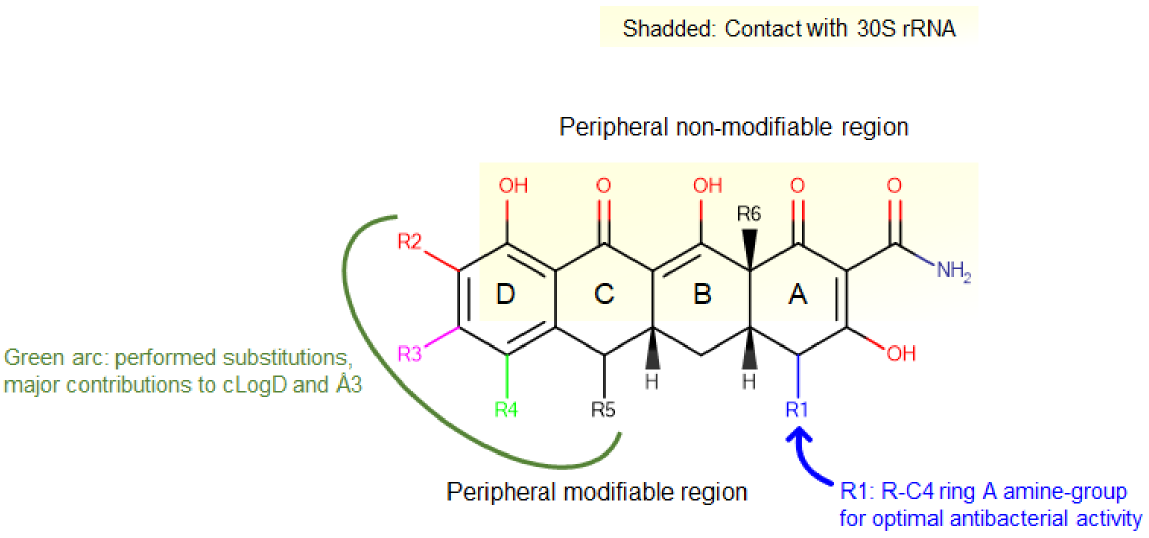

Figure S2. Structure-activity relationship of tetracyclines.

Table S1. List of tetracycline derivatives. Molecules in bold font (**3c**, **3j**, and **3u**) were selected for uptake assays shown in Figure 4 and/or *in vitro* expression assays shown in Figure 5.

Name	n°	MIC (µg/mL)		Fold reduction in MIC	cLogD pH 7.4
		without NV716	with NV716 (10 µM)		
Chlortetracycline	3a	6.25	0.7	9	-4.3
Demeclocycline	3b	6.25	0.4	16	-3.9
**Doxycycline**	**3c**	**12.5**	**0.2**	**63**	**-2.7**
RCT	3d	200	6.25	32	-2.8
RT	3e	400	12.5	32	-2.8
RDOX	3f	400	25	16	-2.7
7-iodo-RDOX	3g	400	6.25	64	-2.5
9-iodo-RDOX	3h	400	12.5	32	-2.8
RDMC	3i	200	6.25	32	-2.4
**Col3**	**3j**	**400**	**3.125**	**128**	**-1.95**
DDOX	3k	400	25	16	-1.8
9-(4-methoxy-phenyl)-RDOX	3l	400	6.25	64	-1.8
9-(3.4-dimethoxyphenyl)-RDOX	3m	400	6.25	64	-1.8
9-(3.4.5-trimethoxyphenyl)-RDOX	3n	400	6.25	64	-1.8
DDMC	3o	250	50	5	-1.9
DCT	3p	400	100	4	-1.6
9-(2-hydroxyphenyl)-RDOX	3q	400	12.5	32	-1.75
9-(3-hydroxyphenyl)-RDOX	3r	400	25	16	-1.75
9-(2-furanyl)-RDOX	3s	400	400	1	-1.4
9-(3.4-methylenedioxyphenyl)-RDOX	3t	400	400	1	-1.2
**-**	**3u**	**400**	**400**	**1**	**-1.08**
(Z)-9-(2-(hydroxymethyl)-hex-1-en-1-yl)-RDOX	3v	400	400	1	-0.9
9-phenyl-RDOX	3w	400	400	1	-0.89
9-tertButyl-Dox-HCl	3x	400	400	1	-0.85
9-(2-thiophenyl)-RDOX	3y	400	400	1	-0.5
9-(2-furanyl)-RDOX	3z	400	400	1	-0.3
9-(2-naphthyl)-RDOX	3aa	400	400	1	0.2
9-(3-benzyloxyphenyl)-RDOX	3ab	400	400	1	0.5
